# Adaptation of WHO COVID-19 guidelines by Caribbean countries and territories

**DOI:** 10.2471/BLT.23.290796

**Published:** 2024-05-23

**Authors:** Tracy Evans-Gilbert, Edmund Blades, Ronela Boodoosingh, Michael H Campbell, Celia DC Christie, Marvin Manzanero, Janice Mullings-George, Earl Ottley, Cil-Maria Outerbridge, Natasha P Sobers, Tamu Davidson, Rian M Extavour, Joy St John, Ludovic Reveiz, Begona Sagastuy, Ignacio Neumann

**Affiliations:** a University of the West Indies, Mona, Kingston, Jamaica.; b Queen Elizabeth Hospital, Bridgetown, Barbados.; c South-West Regional Health Authority, San Fernando, Trinidad and Tobago.; d Belmopan, Belize.; e Ministry of Health, Hamilton, Bermuda.; f North-Central Regional Health Authority, Chaguanas, Trinidad and Tobago.; g Bermuda Hospitals Board, King Edward VII Memorial Hospital, Hamilton, Bermuda; h University of the West Indies, Cave Hill, Barbados.; i Caribbean Public Health Agency, Port of Spain, Trinidad and Tobago.; j Pan American Health Organization, Washington DC, United States of America.; k School of Medicine, Universidad San Sebastian, Lota 2465, Providencia, Santiago 7510157, Chile.

## Abstract

The normative role of the World Health Organization (WHO) involves creating evidence-based, principled guidelines to guide its Member States in making well-informed public health decisions. While these guidelines often need to be adapted to ensure contextual relevance, foster better implementation and adherence, adapting existing guidelines is more efficient than creating new ones. Here we describe the adaptation of the WHO coronavirus disease 2019 (COVID-19) living guideline on pharmacological interventions for the Caribbean using the grading of recommendations, assessment, development and evaluation (GRADE)-ADOLOPMENT method. The Caribbean Public Health Agency and the Pan American Health Organization led the effort, assembling a diverse panel of 16 experts from seven Caribbean countries and territories. The adaptation process, involving 15 steps, was guided by an experienced methodologist and included selecting relevant clinical questions and prioritizing them based on regional needs. The panel evaluated the latest WHO guidelines and integrated additional local data. They adjusted the direction and strength of several recommendations to better fit the Caribbean context, considering local values and preferences, resources, accessibility, feasibility and impact on health equity. Ultimately, we changed the direction of two recommendations and the strength of five, tailoring them to regional realities. This effort highlights the importance of adapting global guidelines to local settings, improving their applicability and effectiveness. The adaptation process also serves as a valuable opportunity for skill transfer and capacity-building in guideline development. Continued research is needed to assess the impact of these adaptations on health-care outcomes in the Caribbean.

## Résumé

Le rôle normatif de l’Organisation mondiale de la santé (OMS) consiste à élaborer des lignes directrices fondées sur des données scientifiques et sur des principes afin d’aider ses États membres à prendre des décisions éclairées en matière de santé publique. Bien qu’elles nécessitent souvent des adaptations pour garantir leur pertinence par rapport à des contextes précis et pour favoriser une meilleure mise en œuvre et adhésion, il est plus efficace d’adapter les lignes directrices existantes que d’en créer de nouvelles. La présente publication décrit l’adaptation de la ligne directrice évolutive de l’OMS sur les interventions pharmacologiques dans le cadre de la maladie à coronavirus (COVID-19) pour les Caraïbes en appliquant la méthodologie GRADE (classement des recommandations, de l’appréciation, du développement et de l’évaluation)-ADOLOPMENT. L’Agence de santé publique des Caraïbes et l’Organisation panaméricaine de la Santé ont dirigé les travaux en réunissant un groupe diversifié de 16 experts issus de sept pays et territoires des Caraïbes. Le processus d’adaptation, comptant 15 étapes et encadré par un méthodologiste expérimenté, a consisté à sélectionner des questions cliniques pertinentes et à les classer par ordre de priorité selon les besoins régionaux. Ce groupe a évalué les dernières lignes directrices de l’OMS et a intégré des données locales supplémentaires. Il a ensuite ajusté l’orientation et le poids de plusieurs recommandations afin de mieux les adapter au contexte des Caraïbes, en tenant compte des valeurs et des préférences locales, des ressources, de l’accessibilité, de la faisabilité et de l’impact sur l’équité en matière de santé. En fin de compte, l’orientation de deux recommandations et le poids de cinq autres ont été modifiés, en les adaptant aux réalités régionales. Cet effort souligne l’importance d’adapter des lignes directrices mondiales aux contextes locaux, afin d’en améliorer l’applicabilité et l’efficacité. Le processus d’adaptation représente également une occasion précieuse de transfert de compétences et de renforcement des capacités en matière d’élaboration de lignes directrices. Des recherches continues s’imposent pour évaluer l’impact de ces adaptations sur les résultats des soins de santé dans les Caraïbes.

## Resumen

La función normativa de la Organización Mundial de la Salud (OMS) consiste en elaborar directrices basadas en pruebas y principios para orientar a sus Estados Miembros en la toma de decisiones de salud pública bien fundamentadas. Aunque con frecuencia es necesario adaptar estas directrices para garantizar su pertinencia contextual y fomentar una mejor implementación y observancia, la adaptación de directrices existentes es más eficiente que la creación de otras nuevas. Aquí describimos la adaptación de la directriz vigente de la OMS sobre la enfermedad por coronavirus (COVID-19) relativa a las intervenciones farmacológicas para el Caribe utilizando el método de clasificación de valoración, elaboración y evaluación de las recomendaciones (GRADE)-ADOLOPMENT. La Agencia de Salud Pública del Caribe y la Organización Panamericana de la Salud lideraron la iniciativa, que reunió a un variado grupo de 16 expertos de siete países y territorios caribeños. El proceso de adaptación, que comprendió 15 pasos y fue guiado por un metodólogo experimentado, incluyó la selección de preguntas clínicas pertinentes y su priorización en función de las necesidades regionales. El grupo evaluó las últimas directrices de la OMS e integró datos locales adicionales. Ajustaron la orientación y la fuerza de varias recomendaciones para adaptarlas mejor al contexto caribeño, teniendo en cuenta los valores y preferencias locales, los recursos, la accesibilidad, la viabilidad y el impacto en la equidad sanitaria. Finalmente, cambiamos la orientación de dos recomendaciones y la fuerza de cinco para adaptarlas a las realidades regionales. Esta iniciativa destaca la importancia de adaptar las directrices mundiales a los contextos locales, lo que mejora su aplicabilidad y eficacia. El proceso de adaptación también constituye una valiosa oportunidad para la transferencia de conocimientos y el desarrollo de capacidades en la elaboración de directrices. Es necesario seguir investigando para evaluar el impacto de estas adaptaciones en los resultados de la atención sanitaria en el Caribe.

## ملخص

يتضمن الدور المعياري لمنظمة الصحة العالمية (WHO) وضع مبادئ توجيهية أساسية قائمة على الأدلة لتوجيه الدول الأعضاء لديها في ما يتعلق باتخاذ قرارات مستنيرة في مجال الصحة العامة. في حين أن هذه المبادئ التوجيهية تحتاج في كثير من الأحيان إلى التكييف لضمان ملاءمتها للسياق، وتعزيز التنفيذ والالتزام بشكل أفضل، إلا إن تكييف المبادئ التوجيهية القائمة يُعد أكثر فعالية من وضع مبادئ توجيهية جديدة. نحن هنا نصف تكييف المبدأ التوجيهي الحالي لدى منظمة الصحة العالمية لمرض فيروس كورونا (كوفيد 19)، والخاص بالتدخلات الدوائية لمنطقة الكاريبي باستخدام طريقة تصنيف التوصيات، والتقويم، والتطوير، والتقييم (GRADE)-ADOLOPMENT. وقامت كل من هيئة الصحة العامة في منطقة الكاريبي، ومنظمة الصحة للدول الأمريكية، بقيادة هذا الجهد، حيث قامتا بتكوين لجنة متنوعة من 16 خبيرا من سبعة من دول وأقاليم منطقة الكاريبي. وتضمنت عملية التكيف 15 خطوة، وتم توجيهها بواسطة منهجية ذات خبرة، وشملت اختيار الأسئلة الإكلينيكية ذات الصلة وتحديد أولوياتها بناءً على الاحتياجات الإقليمية. وقام الفريق بتقييم أحدث المبادئ التوجيهية لمنظمة الصحة العالمية، ودمج البيانات المحلية الإضافية. وقاموا بتعديل اتجاه وقوة العديد من التوصيات لتتناسب بشكل أفضل مع سياق منطقة الكاريبي، مع الأخذ في الاعتبار القيم والتفضيلات المحلية، والموارد، وإمكانية الوصول، والجدوى، والتأثير على المساواة الصحية. وفي النهاية، قمنا بتغيير اتجاه توصيتين وقوة خمس توصيات، حيث قمنا بتخصيصها وفقًا لحالات الواقع الإقليمي. يسلط هذا الجهد الضوء على أهمية تكييف المبادئ التوجيهية العالمية مع الأوضاع المحلية، وتحسين قابلية تطبيقها وفعاليتها. تعد عملية التكيف أيضًا بمثابة فرصة ذات قيمة لنقل المهارات وبناء القدرات في وضع المبادئ التوجيهية. هناك حاجة إلى مواصلة الأبحاث لتقييم تأثير هذه التعديلات على نتائج الرعاية الصحية في منطقة الكاريبي.

## 摘要

世界卫生组织 (WHO) 的规范性作用包括创建有据可依的原则性指南,以指导其成员国在充分了解相关信息的基础上制定公共卫生决策。虽然通常需要调整这些指南来确保符合当地情况,从而有利于更顺利地实施方针和提高遵从性,但是调整现有指南比创建新指南的效率还是更高。在本文中,我们描述了如何使用推荐分级的评估、制定与评价 (GRADE) 工作组开发的 ADOLOPMENT 方法根据加勒比地区的情况对世界卫生组织的冠状病毒病(新冠肺炎)药物干预相关生活指南进行调整。加勒比公共卫生署和泛美卫生组织领导实施该项工作,并从七个加勒比国家和地区邀请了 16 名专家以组建多元化小组。调整过程共分为 15 个步骤,由一位经验丰富的方法学家指导实施,包括选择相关临床问题,并根据区域需求对其进行优先排序。该小组评估了世界卫生组织的最新指南,并综合考虑了其他地方数据。考虑到当地的价值观和偏好、资源、可及性、可行性以及对卫生公平性的影响,该小组调整了几项建议的实施方向和力度,以确保更加符合加勒比地区的情况。最终,我们修改了两项建议的实施方向和五项建议的实施力度,以确保其符合区域实际情况。该项工作突出了根据当地情况调整全球指南、提高其适用性和有效性的重要性。调整过程也是在指南制定过程中实现技能转让和能力建设的宝贵机会。需要继续进行研究,以评估这些调整对加勒比地区医疗保健结果的影响。

## Резюме

Нормативная функция Всемирной организации здравоохранения (ВОЗ) заключается в разработке основанных на фактических данных принципов, которыми должны руководствоваться государства-члены ВОЗ при принятии обоснованных решений в области общественного здравоохранения. Несмотря на то что эти руководящие принципы часто требуют адаптации для обеспечения их соответствия контексту, более эффективного внедрения и соблюдения, адаптация существующих руководящих принципов является более эффективной по сравнению с созданием новых. В данной статье приведено описание адаптации руководящего принципа ВОЗ по фармакологическим вмешательствам при коронавирусной болезни (COVID-19) для стран Карибского бассейна с использованием метода градации рекомендаций, анализа, развития и оценки (GRADE)-ADOLOPMENT. Карибское агентство общественного здравоохранения и Панамериканская организация здравоохранения возглавили эту работу, собрав разнообразную группу из 16 экспертов из семи стран и территорий Карибского бассейна. Процесс адаптации, состоящий из 15 этапов и проводимый под руководством опытного методиста, включал отбор актуальных клинических вопросов и определение их приоритетности с учетом региональных потребностей. Группа экспертов оценила последние рекомендации ВОЗ и включила дополнительные местные данные. Специалисты скорректировали направление и силу некоторых рекомендаций для более полного соответствия условиям Карибского бассейна, учитывая местные ценности и предпочтения, ресурсы, доступность, осуществимость и влияние на справедливость в сфере здравоохранения. В итоге с учетом региональных реалий были изменены направления двух и степень важности пяти рекомендаций. Эти усилия подчеркивают важность адаптации глобальных руководящих принципов к местным условиям, что повышает их применимость и эффективность. Процесс адаптации также представляет собой ценную возможность для передачи навыков и наращивания потенциала в области разработки рекомендаций. Для оценки влияния таких адаптаций на результаты медицинского обслуживания в странах Карибского бассейна необходимо проведение дальнейших исследований.

## Introduction

Although the coronavirus disease 2019 (COVID-19) pandemic was a global catastrophe, its impact was not felt equally around the world. Despite being home to only 8% of the world’s population, Latin America and the Caribbean accounted for approximately 15% of cases and 28% of deaths worldwide.^1^ As of early 2022, 65.4 million cases and 1.7 million deaths had been reported in Latin America and the Caribbean.^1^

The World Health Organization (WHO) developed several formal and informal recommendations to manage individuals with suspected or confirmed COVID-19. One relevant document is the living guideline regarding pharmacological strategies.^2^ Even though WHO guidelines are developed for a global audience, specific constraints of particular health systems may limit their application within certain settings. Indeed, when international guidelines are adapted to specific regions, a significant proportion of recommendations may need to be modified to accommodate the contextual factors of the setting in which they will be applied.^3^ Adaptations may also improve the feasibility of implementation in a local context, and may facilitate acceptance and adherence.^4^

Adapting international guidelines, rather than developing them de novo, has many advantages. Because the development of high-quality evidence-based recommendations is a lengthy and resource-intensive process, adaptation is efficient and helps to avoid unnecessary duplication of work. As well as fostering international collaboration, adaptation also allows for the recommendations to be tailored to the specific circumstances in which they will be implemented, increasing the value and utility of the guideline.

**Fig. 1:**
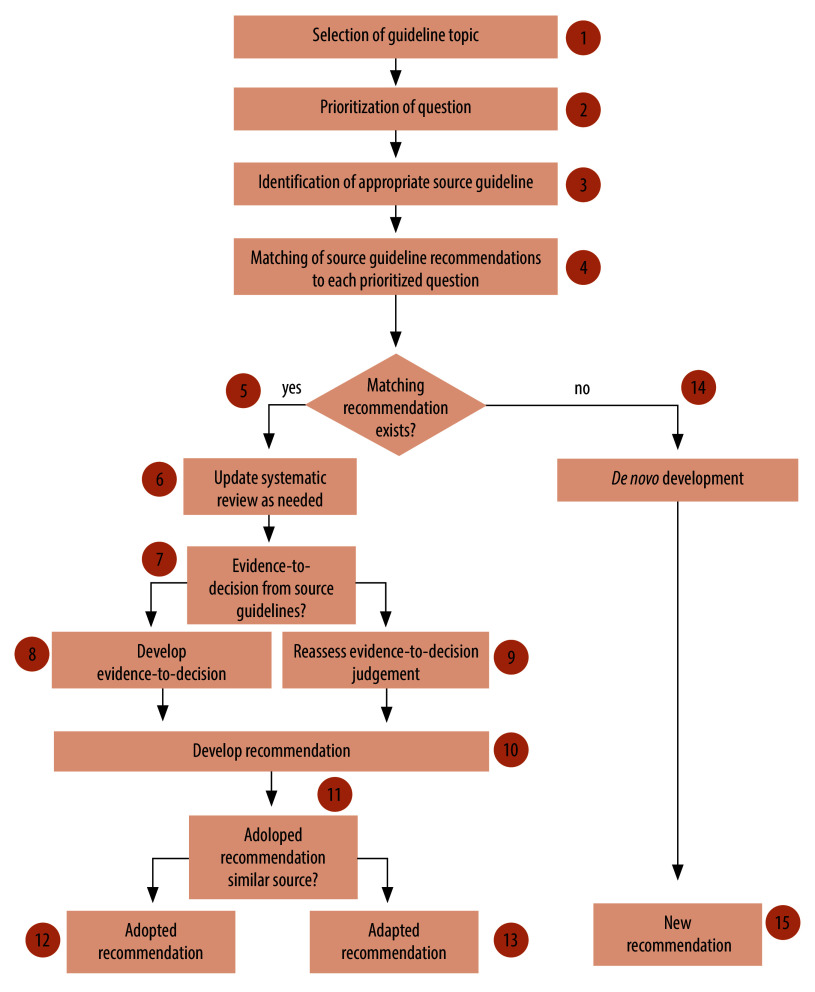
Framework of GRADE-ADOLOPMENT process used to adapt existing guidelines to Caribbean countries and territories, 2022

Although the COVID-19 pandemic has entered a more stable phase, numerous challenges remain in the Caribbean where a large proportion of individuals do not have access to vaccination and early treatment.^5^ A lack of regional Caribbean and local guidance may also have resulted in unwanted variability in the interventions used to treat COVID-19.^1^ We therefore describe the adaptation of the WHO guidelines to the Caribbean using the grading of recommendations, assessment, development and evaluation (GRADE)-ADOLOPMENT method,^6^ and according to the standards adopted by the Institute of Medicine^7^ and the Guideline International Network.^8^ This approach is based on the principle of reusing the evidence identified by international guidelines, but adding relevant local data and revisiting the judgements that determined the direction and strength of the recommendations from a local perspective.^9^ The 15-step adaptation process, a joint effort of the Caribbean Public Health Agency and the Pan American Health Organization (PAHO), includes selection of guideline topic and prioritization of questions using the GRADE evidence-to-decision framework (Fig. 1).^10^

GRADE: grading of recommendations, assessment, development and evaluation.

Source: Adapted from World Health Organization.^9^

## Methods

### Panel selection

The Caribbean Public Health Agency, representing 24 countries and territories^11^, invited 42 nominees from its members to participate as guideline panellists. The agency considered geographical representation and gender diversity when identifying nominees, and accepted 16 nominees from seven countries and territories (Barbados; Belize; Bermuda; Jamaica; Montserrat; Trinidad and Tobago; and Turks and Caicos). One person who was in the process of completing a training course on guideline development was invited to join as an observer. The agency proposed one of these 16 nominees to serve as the guideline clinical chair. PAHO identified and recommended an experienced guideline methodologist to serve as the guideline methods chair. Consequently, our panel comprised a clinical chair, a methods chair, 12 content experts, and a representative from each of the Caribbean Public Health Agency and PAHO. Of the 16 panel members, three were medical officers from the health ministries in Bermuda, Trinidad and Tobago, and the Turks and Caicos Islands. The 13 content experts, who also represented the intended end-users of the guideline, comprised health-care professionals from a broad spectrum of disciplines (frontline physicians, infectious disease specialists, microbiologists, pharmacists, nurses, a behavioural scientist and a psychologist).

The clinical chair and two other panellists had previous experience of developing recommendations, but none of the panellists had developed or adapted guidelines using the GRADE method before. The methods chair possessed significant expertise in formulating guidelines using the GRADE method, and had previously conducted successful regional adaptations within Latin America.^3^^,^^12^^–^^14^ The methods chair delivered a training session on guideline methods to the other panellists before issuing recommendations.

We managed panellist conflicts of interest according to recommendations of the Institute of Medicine and the Guidelines International Network.^7^^,^^8^ Participants disclosed all financial and nonfinancial interests relevant to the guideline topic. Caribbean Public Health Agency staff (non-panellists) reviewed the disclosures and made judgments about conflicts. We ensured there were no conflicts that debarred panellists from participating in the guideline adaptation process, including direct financial conflicts with for-profit companies.

### Selecting guideline topic and source 

The GRADE-ADOLOPMENT process begins with the selection of a guideline topic, as outlined in step 1 of Fig. 1. This is typically followed by prioritization of questions (step 2) before identification of an appropriate source guideline (step 3). However, the current adaptation occurred during the COVID-19 pandemic, a period characterized by the introduction of additional therapeutic options for wider use and challenges in prioritizing resources within health systems. We therefore recognized the global WHO COVID-19 living guideline^2^ – a comprehensive and ongoing effort that includes many relevant interventions for managing COVID-19 – as an appropriate and complete source of material, justifying its selection before the prioritization of questions.

### Prioritization of questions 

This critical stage of the adolopment process involves identifying the relevant clinical questions and matching them with the source guidelines (steps 2 and 5 in Fig. 1). During an online meeting, our guideline panel selected 13 relevant questions (Table 1) covered by the source guideline^2^ through discussion and consensus based on three criteria, namely those: (i) that commonly arise in practice in the context of the Caribbean; (ii) associated with variation in practice across countries and territories in the Caribbean; and (iii) that may be associated with high resource use or costs. Our panellists then prioritized the relevant outcomes using a 9-point scale and rated them as either critical (score 7–9), important (score 4–6) or less important (score 1–3) for decision-making. We considered the critical outcomes of mortality, mechanical ventilation, hospitalization or adverse events in the adaptation process.

**Table 1:** Questions covered by WHO guideline on drugs for COVID-19^2^ considered for potential adaptation via GRADE-ADOLOPMENT for Caribbean countries and territories, 2022

Prioritized questions, including the target population and intervention of interest	WHO recommendation^2^	Adaptation for Caribbean countries and territories
**Non-severe COVID-19**		
1. In individuals with non-severe COVID-19 and low risk of complications, should we use nirmatrelvir-ritonavir in addition to standard treatment?	Conditional recommendation against	Unchanged
2. In individuals with non-severe COVID-19 and high risk of complications, should we use nirmatrelvir-ritonavir in addition to standard treatment?	Strong recommendation in favour of	Conditional recommendation in favour of
3. In individuals with non-severe COVID-19 and low risk of complications, should we use molnupiravir in addition to standard treatment?	Conditional recommendation against	Unchanged
4. In individuals with non-severe COVID-19 and high risk of complications, should we use molnupiravir in addition to standard treatment?	Conditional recommendation in favour of	Conditional recommendation against
5. In individuals with non-severe COVID-19 and low risk of complications, should we use remdesivir in addition to standard treatment?	Conditional recommendation against	Unchanged
6. In individuals with non-severe COVID-19 and high risk of complications, should we use remdesivir in addition to standard treatment?	Conditional recommendation in favour of	Conditional recommendation against
7. In individuals with non-severe COVID-19 and should we use systemic corticosteroids in addition to standard treatment?	Conditional recommendation against	Strong recommendation against
**Severe and critical COVID-19**		
8. In individuals with severe and critical COVID-19, should we use systemic corticosteroids as part of the standard treatment?	Strong recommendation in favour of	Unchanged
9. In individuals with severe and critical COVID-19, should we use IL-6 blockers as part of the standard treatment?	Strong recommendation in favour of	Conditional recommendation in favour of
10. In individuals with severe and critical COVID-19, should we use baricitinib as part of the standard treatment?	Strong recommendation in favour of	Conditional recommendation in favour of
11. In individuals with severe and critical COVID-19, should we use remdesivir as part of the standard treatment?	Conditional recommendation in favour of	Unchanged
**General recommendations (any severity)**		
12. In individuals with COVID-19 (any severity), should we use hydroxychloroquine in addition to standard treatment?	Strong recommendation against	Unchanged
13. In individuals with COVID-19 (any severity), should we use ivermectin in addition to standard treatment?	Conditional recommendation against	Strong recommendation against

COVID-19: coronavirus disease 2019; GRADE: grading of recommendations, assessment, development and evaluation; IL-6: interleukin-6; WHO: World Health Organization.

### Evidence reviews and local data

After selecting and prioritizing questions, the next stage of the adolopment process is to assess whether the source evidence is up to date (step 6 in Fig. 1). For each of the questions selected during the previous stage (Table 1), the methodologist assessed the evidence presented in the latest version of the WHO COVID-19 living guideline (October 2022).^2^ Given the living nature of the source guideline, we did not consider that an update of the source systematic reviews was necessary. However, the source guideline lacked the evidence-to-decision framework that typically accompanies GRADE recommendations. The methodologist therefore developed an evidence-to-decision framework based on both the information available in the source guideline and additional data that we gathered for this purpose (steps 7–9 in Fig. 1). 

For this adaptation, we incorporated the latest GRADE guidance using predefined thresholds for judging imprecision. We developed these thresholds during an online meeting by discussing and analysing results from an online survey of the panellists conducted by the methods chair. For all critical outcomes, we asked panellists to judge the threshold between a trivial to small benefit, a small to moderate benefit and a moderate to large benefit.^15^^–^^17^ The methods chair also surveyed the panellists to collect data regarding the estimated cost of the different options, as well as potential barriers to accessibility and affordability in the different countries and territories across the Caribbean. We summarized the data for each question in an evidence-to-decision table^10^ using the GRADEpro Guideline Development Tool (McMaster University, Hamilton, Canada; Evidence Prime, Inc., Kraków, Poland).

### Development of recommendations

Our final stage was the development of recommendations using the updated evidence-to-decision framework (steps 11–13 in Fig. 1). Rather than presenting the final recommendation from the source guideline, we tasked panellists with critically assessing evidence from both the source guideline and relevant, context-specific data. The panel were then required to make explicit judgements with regards to the size of benefits and harms; the certainty of the evidence; the variability of patients’ values and preferences; and contextual factors such as the resources required to implement the intervention, the potential impact on health equity, and the acceptability and feasibility of implementing the intervention.

During three online meetings (15 and 29 September, 13 October 2022), our panel assessed the updated evidence-to-decision frameworks (which included context-specific data) and made judgements with regards to each individual domain of the evidence-to-decision framework. Panellists agreed on such judgments by consensus through group discussion and deliberation. In rare instances when consensus was not reached, we conducted a vote. From these judgements, the panel developed recommendations specifying the direction and strength of each recommendation, along with a rating of the certainty of the evidence. 

We published the adapted recommendations online, along with the evidence-to-decision frameworks, in December 2022; we also published the adapted recommendations in the large RecMap database of COVID-19 recommendations.^18^^,^^19^

## Results

Of the 13 recommendations considered from the original WHO guideline, the panellists changed the direction of two and the strength of five. We list the seven adapted and six unchanged recommendations in Table 1. We provide the complete list of recommendations for the Caribbean and a summary infographic in the online supplementary file.^20^

### Changes in recommendation direction 

Our panel changed two recommendations from conditional in favour of to conditional against (questions 4 and 6). Both recommendations assessed the use of antivirals in patients with non-severe COVID-19 but with a relatively high risk of hospitalization (> 10%). These recommendations concerned the drugs molnupiravir and remdesivir. Because the panel considered that the benefit of either of these antivirals was relatively small in absolute terms, combined with significant barriers to accessibility and affordability in the Caribbean, implementing these interventions may have induced a negative impact on health equity. Most importantly, the potentially more effective alternative of nirmatrelvir-ritonavir is more readily available in some countries in the Caribbean. Also, for those countries in which antivirals are not yet available, reducing the number of options to those that appear to be more effective may allow resources to be more efficiently allocated.

### Changes in recommendation strength 

Our panel changed three recommendations from strong in favour of to conditional in favour of (questions 2, 9 and 10). The first recommendation assessed the effect of nirmatrelvir-ritonavir in patients with non-severe COVID-19 but with a high risk of hospitalization (> 10%). The other two recommendations evaluated the use of interleukin-6 blockers and the use of baricitinib in individuals with severe and critical COVID-19. The panellists considered that the benefits of the intervention in all three recommendations were relatively small, and identified important barriers to accessibility, affordability and feasibility. In the context of constrained resources, the panel also considered that the use of costly interventions should be prioritized for those with greater needs to minimize the negative impact on health equity. We considered these arguments to fit better with a conditional than a strong recommendation.

Our panel also changed two recommendations from conditional against to strong against (questions 7 and 13). One recommendation assessed the use of systemic corticosteroids in non-severe COVID-19, and the other the use of ivermectin in COVID-19 of any severity. In both cases, the panel considered that there was no appreciable benefit from the intervention, but there was moderate certainty of harm.

## Discussion

Guideline development is a lengthy and resource-intensive process. Because most of the high-quality guidelines are developed in high-income countries, the particularities of low- and middle-income settings are typically underrepresented in the international guideline development process. In addition, the knowledge and skills required to produce high-quality guidelines are also concentrated in high-income countries. These factors combine to create a situation in which settings such as in Latin America and the Caribbean are not well represented in the production of international guidelines; professionals from these regions also have a limited capacity to develop their own evidence-based recommendations.

Adaptation processes as described here may help to reduce the knowledge gap between high-income countries and low- and middle-income countries. The process itself also creates an opportunity to transfer skills and knowledge in guideline development. Notably, none of the panellists who participated in this adaptation had any previous experience with GRADE guidelines; however, with the collaboration of international methodologists experienced in guideline development, our panel was able to produce high-quality recommendations according to the standards promoted by the Institute of Medicine^7^ and the Guideline International Network.^8^

The work reported in this paper aligns with WHO and PAHO plans to promote contextualization of global guidelines to local settings using clear and transparent methods, such as the adolopment method. In 2018, PAHO published a tool for adapting and implementing guidelines in the Region of the Americas^21^ that was subsequently updated in 2023.^22^ At the end of 2023, a second handbook was published by the WHO Regional Office for Europe.^9^ These documents encapsulate the knowledge acquired from a decade of adaptation initiatives, through specific regional projects and technological tools designed to facilitate and champion the processes of adaptation and contextualization.^23^ A significant update to the WHO Handbook for Guideline Development^24^ is anticipated by the end of 2024, expected to include detailed methods for adapting and contextualizing guidelines. In this new model, global guidelines may serve as a foundational framework, enabling regional offices or Member State governments to efficiently develop customized recommendations that specifically address their unique needs.

Despite its benefits, the adaptation process presents notable challenges. Primarily, it demands additional time and effort beyond adopting a global recommendation. Regardless, evidence indicates that recommendations change by as much as 30% when tailored to specific contexts,^12^^–^^14^ suggesting that the extra investment could lead to significantly enhanced recommendation applicability. 

Second, even with explicit methods such as adolopment, the adaptation process demands the involvement of methodologists and content experts who possess knowledge and experience of guideline development. Without a robust monitoring process, there is a risk that adaptations could yield recommendations of diminished quality, that might not be based on the underlying evidence or are compromised by conflicts of interest. In this context, engaging in a transparent process such as described here could contribute to building the essential skills needed for the effective contextualization of WHO guidelines. 

Third, the creation of global guidelines and their contextualization or adaptation often occur as separate initiatives; this division of processes frequently means that the specific needs of particular regions or Member States are not adequately addressed by global guidelines. Indeed, panellists participating in this adaptation identified several topics that were not addressed in detail in the original WHO guideline and were therefore not available for adaptation. The more important clinical questions identified included those related to the management of post-COVID-19 conditions. International collaboration and integration between global developers and regional offices are important to foster coordinated efforts.

We have highlighted the need to contextualize recommendations for the settings in which they are going to be applied. Adapting international COVID-19 guidelines to the Caribbean countries and territories resulted in important changes to the recommendations. Further research is required to assess the final outcomes of the adaptation efforts. The degree to which tailored and contextualized recommendations enhance adoption and promote a health-care system of excellence, attuned to the differences in settings and patient values, remains an area for exploration. 
